# Identification of potential leads against 4-hydroxytetrahydrodipicolinate synthase from Mycobacterium tuberculosis

**DOI:** 10.6026/97320630012400

**Published:** 2016-12-02

**Authors:** Ajijur Rehman, Salman Akhtar, Mohd Haris Siddiqui, Usman Sayeed, Syed Sayeed Ahmad, Jamal M. Arif, M. Kalim A. Khan

**Affiliations:** 1 Department of Biosciences, Faculty of Applied Sciences, Integral University Lucknow, Uttar Pradesh, India-226026; 2 Department of Bioengineering, Faculty of Engineering, Integral University Lucknow, Uttar Pradesh, India-226026

**Keywords:** DHDPS, Mycobacterium tuberculosis, docking, phyto-compound, drug-likeness

## Abstract

4-hydroxy-tetrahydrodipicolinate synthase (DHDPS) is an important enzyme needed for the biosynthesis of lysine and many more key metabolites in Mycobacterium tuberculosis (Mtb). Inhibition of DHDPS is supposed to a promising therapeutic target due to its specific role in sporulation, cross-linking of the peptidiglycan polymers and biosynthesis of amino acids. In this work, a known inhibitor-based similarity search was carried out against a natural products database (Super Natural II) towards identification of more potent phyto-inhibitors. Molecular interaction studies were accomplished using three different tools to understand and establish the participation of active site residues as the key players in stabilizing the binding mode of ligands and target protein. The best phyto-compound deduced on the basis of binding affinity was further used as a template to make similarity scan across the PubChem Compound database (score > = 80 %) to get more divesred leads. In this search 5098 hits were obtained that further reduced to 262 after drug-likeness filtration. These phytochemicallike compounds were docked at the active site of DHDPS.Then, those hits selected from docking analysis that showing stronger binding and forming maximum H-bonds with the active site residues (Thr54, Thr55, Tyr143, Arg148 and Lys171). Finally, we predicted one phytochemical compound (SN00003544), two PubChem-compounds (CID41032023, CID54025334) akin to phytochemical molecule showing better interactions in comaprison of known inhibitors of target protein.These findings might be further useful to gain the structural insight into the designing of novel leads against DapA family.

## Background

Mycobacterium tuberculosis (Mtb), a brutal killer of the human population by spreading most infectious disease, tuberculosis (TB) has been avowed a big threat to public health across the globe [1]. The Global Tuberculosis Control 2015 has mentioned the statistics regarding the occurrence of 9.6 million of new cases (and 1.5 million patients deaths from TB in the year 2014, out of which 12% of the new cases were HIV-positive patient [1].The year 2015 is seen for a defining moment in the battling against TB where move has been begun from the Millennium Development Goals (MDGs) to another age of Sustainable Development Goals (SDGs), and a step ahead towards complete eradication of this disease [1]. With the advancement of technology new TB medications are presently emerging, and combination of different new compounds and even few vaccines are being tested in different phases of clinical trials. Nevertheless, availability of such kind of medication, resistance to the 'isoniazid', 'rifampicin', 'fluoroquinolone' and few second-line injectable drugs is considered one of the biggest hurdles in the way of SDGs [1,2]. Therefore, deciphering potent and effective molecular drug target enzymes for the development of new novel inhibitors with no pre-existing resistance mechanisms is an important emphasis of research.

**Figure 1:**
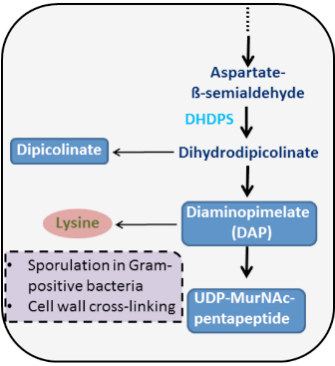
A portion of DAP/Lysine Pathway.

The 4-hydroxy-tetrahydrodipicolinate synthase (P9WP25) is a key enzyme of Lysine/DHDPS biosynthetic pathway of Mtb responsible for synthesis of D, L diaminopimelic acid (meso- DHDPS) and lysine [3,4]. Apart from both components, few important metabolites viz. dihydrodipicolinate, a precursor of dipicolinate and UDP-MurNAc-pentapetide is also produced (Figure 1). Both pathway specific metabolites are respectively essential for sporulation and peptidoglycan cross-linking via covalent interaction with D-alanyl moieties of vicinal chain to generate murein polymers providing stability and rigidity to the bacterial cell wall [3, 4,5]. It has experimentally shown that de novo biosynthesis of lysine is required for the survival of Mtb during infection, albeit its adequacy in the host. Inhibition of Lysine/DHDPS pathway is fatal to the survival of Mtb [3]. Therefore identification of effective inhibitors against enzymes of this pathway should provide leads for the development of new anti-TB drugs.

DHDPS is an important enzyme of lysine biosynthesis pathway that catalyses the condensation of aspartate-β-semialdehyde and pyruvate to 4-hydroxy-tetrahydrodipicolinate (HTPA). Dihydropicolinate (DHDP) is released from active site (Lys-171) with the elimination of water molecule [4]. Structurally DHDPS is a homotetramer enzyme made up of 2 monomers that includes 2 domains (8-fold alpha-/beta-barrel, C-terminal alpha-helical domain). The barrel domain is occupied by the active site residue lysine-171 that has accessibility on the C-terminal of the barrel via 2 entry points.

Numerous inhibitors against Mtb-DHDPS have been identified so for, but quest to find the best is still unexplored. Towards this direction a comparison between experimentally known and predicted inhibitor was made by Garg et al., 2010 through molecular dynamics simulation study. They proposed that PUB475318 is bestowed better inhibition potential as compared to the previously reported inhibitors of Mtb-DHDPS. Keeping these facts on consideration we have used it as a template for search and identification of novel phyto-ligands and diversified PubChem compounds instead of considering experimentally known inhibitors as template.

In the proposed study three different computational tools (e.g., BioPredicta, Molegro Virtual Docker (MVD), and AutoDock Tools) [6,7] was used to decipher potential anti-tubercular leads in terms of better binding energy and inhibition constant [8, 9,10,11] through virtual screening of plant-derived natural compounds database, Super Natural II comprises of about 325,508 molecules and PubChem Compound database of NCBI [4,12]. This work of identifying potent inhibitors of DHDPS is based on rigorous docking analysis of different scoring functions of adopted computational tools yielding the most reliable, consistent and accurate results [6,7]. These findings of proposed study would be a great help to wet-lab biology and computer-aided designing of effective drugs against the most infectious malady.

## Methodology

### Retrieval of protein 3D structure

The crystal structure (3D) of Mtb-DHDPS (PDB ID: 1XXX) was extracted from RCSB Protein Data Bank. The coordinates of the chloride ion, magnesium ion, 2, 3-dihydroxy-1, 4-dithiobutane (DDT), and water molecules were removed to prepare the protein for molecular docking. The protein was energetically minimized using the CHARMm force field.

### Retrieval of ligands 3D structure

**Figure 2:**
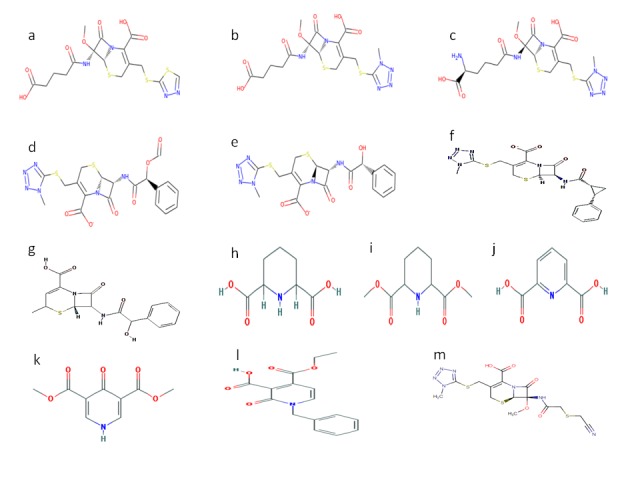
Chemical structyre of ligands- a: SN00234301, b: SN00299194, c: SN00241540, d: SN00074285, e: SN00003544, f: CID41032023, g; CID54025334, h: CID557515, i: CID12265924, j: CID10367, k: CID11390199, l: CID68297515, m: PUB475318

The structure of PUB475318, a newly predicted inhibitor of DHDPS [4], and phyto-compound (SN00003544)-like ligands were obtained from the PubChem database of NCBI. The structures of PUB475318- based similar phytochemicals were extracted from the Super Natural II database (http://www.uefs.br) . By applying CHARMm force, ligands were energetically minimized using the steepest descent algorithm for 500 steps at an RMS gradient of 0.01. Chemical structure of all ligands are shown in Figure 2.

### Drug-likeness prediction

Lipinski rule of five (RO5) was employed to predict the druglikeness of ligands. RO5 includes molecular mass (< = 500 Dalton), high lipophilicity (Log p < = 5) H-bond donors (< = 5), H-bond acceptors (< = 10) and molar refractivity (40-130). These filtrations ensure drug-likeness for molecules obeying two or more features of RO5 [13,14].

### Docking simulation

BioPredicta tool of VlifeMDS package [6], MVD (http://www.clcbio.com) and AutoDock Tools 4.0 were used for molecular interaction studies of ligands and protein.

### BioPredicta

It employed Genetic algorithm (GA), Piecewise Linear Pairwise Potential (PLP) and Grid algorithms energy minimization by using MMFF force fields. The Dock scoring function was used to assess the binding efficacies of ligands. This scoring function take into account the terms for van der Walls interaction, hydrophobic effects, hydrogen bonding and deformation penalty. BioPredicta tool uses following fitness function for searching rigid docking space.

E = InterEq; E = InterEvdW + InterEq; E = EEPIC; Where, InterEq = intermolecular electrostatic energy of complex; InterEvdW = intermolecular vdW energy of complex; EEPIC = electrostatic potential for intermolecular complex

All other required parameters were set as default during the process of molecular interactions.

### MVD

It integrates highly efficient PLP and MolDock scoring function for molecular docking. Docking parameters and other required parameters were set to default values [15]. MolDock-rerank score was further employed to judge the binding affinity of ligands.

### AutoDock

Polar H-atoms, Kollman united atom and atom type parameters were added and further, non-polar H-atoms were merged during generation of the protein pdbqt file. During preparation of ligand pdbqt file, polar H-atoms added, non-polar H-atoms merged, number of torsions, and rotatable bonds were defined. Cubic volume of 40 × 40 × 40 Å 3 with 0.408 Å grid points spacing and X: 3.163, Y: 39.286, Z: 70.258 centre coordinates was set to cover the entire active site and accommodate ligand to move freely. Lamarckian genetic algorithm was employed for the receptor-fixed ligand-flexible docking calculations. The conformer having lowest free energy of binding (ΔG) was considered for further analysis [8, 9,10,11].

## Results and Discussion

**Figure 3:**
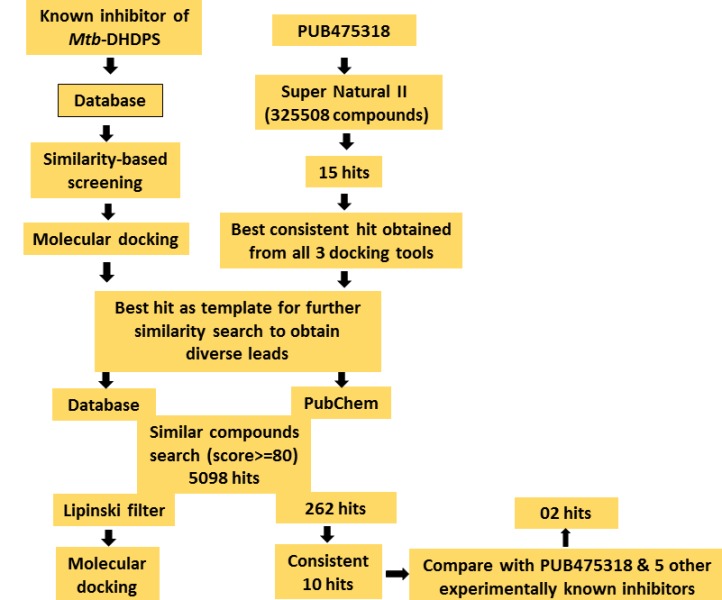
Flowchart of the virtual screening results.

Two approaches were implemented to search and find out the potent leads against Mtb-DHDPS. Virtual screening of phytocompounds from the natural products database of the UEFS (http://www.uefs.br) was performed as first approach using recently predicted inhibitor, PUB475318 as template [4]. In the second approach, similarity search for diverse classes of compounds from the PubChem database were carried out using SN00003544 of the first approach as a template (Figure 3).

### Docking of phyto-compounds

**Figure 4:**
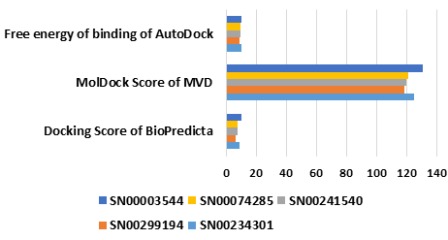
Docking comparison of top phyto-ligands.

**Figure 5:**
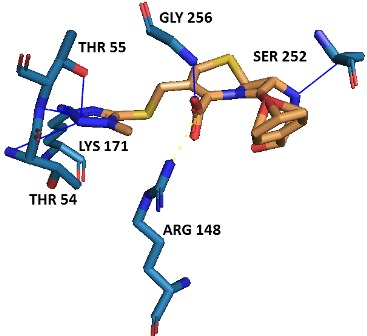
Docking of best phyto-lead (SN00003544) to the active site of Mtb-DHDPS. H-bonds and salt bridge are respectively shown by blue and yellow lines.

Among all phyto-compounds docked with the Mtb-DHDPS, SN00003544 was found to bind with the best efficacy in the Nterminal (β/α)8-barrel domain of Mtb-DHDPS comprises of 1-233 residues [4] as consistently reflected by scoring functions of adopted docking tools (Figure 4). Ala18, Thr54, Thr55, Tyr143, Arg148, Lys171, Ala173, Gly194, Asp195, Asp196, Ile211, and Val213 residues of N-terminal (β/α)8-barrel domain and Met251, Ser252, Gly255, and Gly256 residues of C-terminal alpha-helical domain of target protein were engaged in molecular interactions (Table 1). Among all residues, the active site residues Thr54, Thr55, andLys171 of N-terminal domain and Ser252, and Gly256of Cterminal were engaged in hydrogen bond formation with the best phyto-lead (SN00003544). Hydrogen bonding between DHDPS and SN00003544 provides a directionality and specificity of interaction. Furthermore, Arg148 of N-terminal is also involved in salt bridge formation and thus contributing to protein-ligand stabilization (Figure 5, Table 2). Interaction profiling of ligand and protein in the study was carried out by using PLIP tool [16].

### Docking study of PubChem compounds and their comparison with knwon inhibitors

To search and identify a diverse classes of ligands having antitubercular potential, 3-D similary search (similarity score > = 80 %) against the PubChem compound database was carried out using best phyto-ligand (SN00003544) as template. Initially SN00003544- akin 5098 compounds were retrieved. These molecules were subjected to RO5 filtration before going for docking studies. The 268 molecules out of 5098 were succeeded the RO5 filtration. Molecular docking studies of these compounds were performed for the best binding orientation prediction into the active site of Mtb- DHDPS using the same docking procedure and parameters as mentioned earlier for phyto-compounds. Out of 268 only 50 compounds exhibited plausible binding along with the formation of H-bond with the active site residue Lys171 of Mtb-DHDPS. Further, out of 50 only 10 ligands were observed consistent as bestowed by all three adopted computational tools [6, 7,14]. Similar to the best phyto-lead, H-bonding was found to be more prominent interactions withThr55, Arg148, Lys171, and Gly256 residues. The remaining 218 out of 268 compounds exhibited feeble molecular interactions and also failed to form H-bond with the active site residue Lys171, depicting their least antitubercular potential.

**Figure 6:**
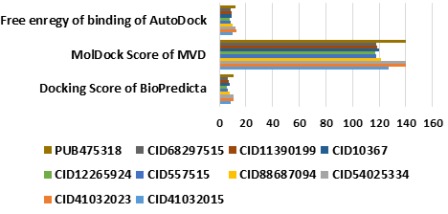
Docking comparison of top PubChem hits with experimentally known and predicted inhibitors

A comparison of top 10 PubChem hits were made with the five experimentally known inhibitors for example piperidine-2,6- dicarboxylic acid (CID557515), dimethylpiperidine-2,6- dicarboxylate (CID12265924), pyridine-2,6-dicarbxylic acid (CID10367), 1,4-dihydro-4-oxopyridine-2,6-dicarboxylic acid (CID11390199), and dimethyl-1,4-dihydro-4-oxopyridine-2,6- dicarboxylate (CID68297515), and a novel predicted inhibitor PUB475318 of Mtb-DHDPS in order to screen the best phyto-leadlike chemical agents. Only 4 out of 10 hits exhibited stronger binding affinity in comparison of 5 experimentally known inhibitors. Furthermore, out of four only two compounds (CID54025334 and CID41032023) were depicted as stronger inhibitors in comparison of PUB475318 as shown by scoring functions of adopted docking tools (Figure 6). Docking scores, hydrogen bonding residues, residues involved in molecular interactions of top four PubChem hits and known inhibitors are summarized in Table 3.

**Figure 7:**
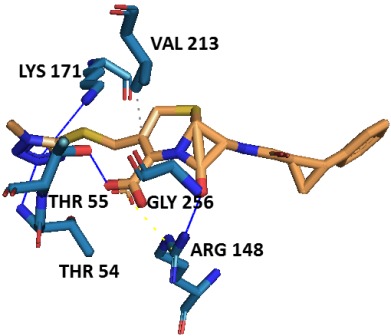
Docking of first potent PubChem compound (CID41032023) to the active site of Mtb-DHDPS. H-bonds, salt bridge, and hydrophobic interaction are respectively shown by blue, yellow, and grey lines.

The active site residues Thr54, Thr55, Arg148, Lys171 of N-terminal domain and Gly256 of C-terminal domain were stabilized the molecular interaction of first potent PubChem ligand (CID41032023) and protein (Mtb-DHDPS) through hydrogen bond formation. Apart from H-bonding, Arg148 is also engaged in salt bridge formation and enhancing the stability of complex (Table 4). Furthermore, Val213 of N-terminal barrel domain was involved in hydrophobic interaction showing energetically favourable association of non polar surfaces of ligand and protein [17] (Figure 7).

**Figure 8:**
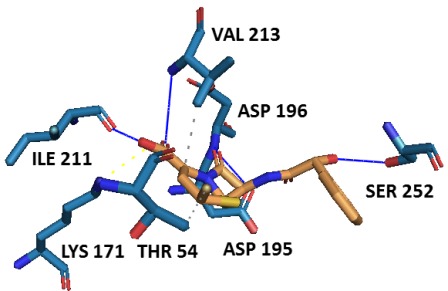
Docking of second potent PubChem compound (CID54025334) to the active site of Mtb-DHDPS. H-bonds, salt bridge, and hydrophobic interaction are respectively shown by blue, yellow, and grey lines.

**Figure 9:**
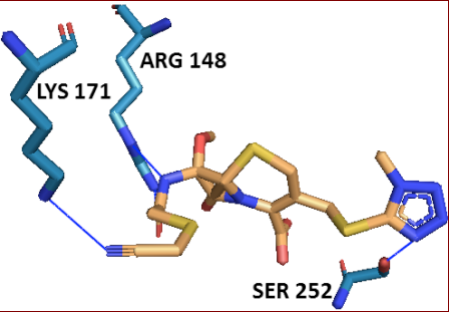
Docking of known inhibitor (PUB475318) to the active site of Mtb-DHDPS. H-bonds, are shown by blue lines.

Likewise, higher binding affinity of second potent PubChem compound (CID54025334) towards Mtb-DHDPS was attributed by the five hydrogen bonding (Asp195, Asp196, Ile211, Val213, and Ser252), two hydrophobic interactions (Thr54, and Val213), and one salt bridge formation (Lys171) (Figure 8, Table 5) demonstrating stronger inhibitory potential of ligand in comparison of known inhibitor (PUB475318). Docking complex of known inhibitor and Mtb- DHDPS and their binding pattern are respectively shown in Figure 9 and Table 6.

### Validation of docking protocol

**Figure 10:**
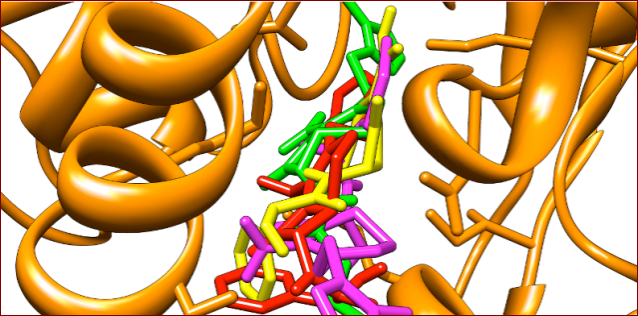
Superimposition of ligands and known inhibitor into the binding cavity of Mtb-DHDPS. SN00003544, CID41032023, CID54025334, and PUB475318 are respectively shown in red, green, yellow, and magenta colors.

Validation of docking procedure adopted in the study was accomplished by superimposing all the ligands showing stronger binding activity into the active site of Mtb-DHDPS [18, 19,20]. The best phyto-lead (SN00003544), phyto-lead like PubChem compounds (CID41032023 and CID54025334) and known inhibitor (PUB475318) were docked into the same binding orientation of target protein and thus favoring the adopted docking procedure (Figure 10).

## Conclusion

In the present study, we employed two virtual screening approaches towards the identification and elucidation of novel drug leads against one of the oldest malady of humankind. In the first approach we screened out a potent natural compound (SN00003544) from the UEFS (http://www.uefs.br) database that bestowed strong binding affinity with Mtb-DHDPS as shown by five hydrogen bonding (Thr54, Thr55, Lys171, Ser252, and Gly256) and one slat bridge formation (Arg148). In the second approach two compounds (CID41032023, CID54025334) akin to phyto-lead with extremely different scaffold from template molecule were identified. These two compounds demonstrated better binding mode into the active site of Mtb-DHDPS and establishing strong bonded and non-bonded molecular interactions (e.g.; hydrogen bonds, salt bridges and hydrophobic interactions) as compared by known inhibitors. In hydrogen bonding distance between donor and acceptor atoms (<4.1 Å), and angle between donor, acceptor and hydrogen atoms (>100°) were found in significant range. Similarly in salt bridges, distance between centers of charge (<5.5 Å) and in hydrophobic interactions, distance between interactions carbon atoms (<4.0 Å) were found significant [16]. Since all three leads predicted in the study have ability to inhibit the activity of target protein by blocking the active site residues via three different important interacting forces (viz. H-bond, salt bridge, and hydrophobic interaction) that determine the stability of biomolecular interactions. Due to strong blockage of active site residues of target protein, de novo biosynthesis of lysine and other secondary metabolites might be impeded during infection and survival of the pathogen threatened. Albeit the wet-lab studies are indispensable to validate the in silico findings of the study, however, predicted leads would certainly help the experimental designing of more potent anti-tubercular agents.

## Conflict of Interest

Authors would like to declare no conflict of interest.

## Figures and Tables

**Table 1:** Molecular interaction studies of top five screened phyto-compounds

S. No.	Molecule ID	Scoring functions a^*^, b^$^, c^^^	H bonding residues	Residues involved in molecular interactions
1	SN00234301	-8.697655	Thr55, Arg148, Lys171, Gly256, Asp195, Met251	Ala18,Thr54,Thr55,Tyr143, Ile145, Gly147, Arg148, Lys171, Gly194, Asp195, Asp196, Ala197, Val213, Cys248 ,Met251, Ser252, Gly255, Gly256
		-124.921		
		-9.98		
2	SN00299194	-6.222914	Thr54, Thr55, Arg148, Asp195	Ala18, Met19,Val50, Gly53, Thr54, Thr55, Gly56, Leu111, Tyr143, Ile145, Arg148, Lys171, Ala173, Gly194, Asp195, Asp196, Val213, Met251
		-118.309		
		-8.85		
3	SN00241540	-7.727655	Thr55, Arg148, Lys171, Gly256	Ala18,Thr54,Thr55,Tyr143, Arg148, Lys171, Gly194, Asp195, Asp196, Ala197, Val213, Met251, Ser252, Gly255, Gly256
		-120.119		
		-9.76		
4	SN00074285	-7.971286	Gly147, Arg148, Lys171, Gly256, Asp195	Ala18,Thr54,Thr55,Tyr143, Ile145, Pro146, Gly147, Arg148, Lys171, Ala173, Lys174, Gly194, Asp195, Asp196, Ala197, Ile211, Val213, Cys248, Met251, Ser252, Gly255, Gly256
		-121.431		
		-9.89		
5	SN00003544	-9.976235	Thr54, Thr55, Arg148, Lys171, Gly256, Ser252	Ala18, Thr54, Thr55, Tyr143, Arg148, Lys171, Ala173, Gly194, Asp195, Asp196, Ile211, Val213, Met251, Ser252, Gly255,Gly256,
		-130.632		
		-10.59		

^*^a: Docking Score of BioPredicta, ^$^b: MolDock Score of MVD, ^^^c: Free energy of binding of AutoDock				

**Table 2:** Binding pattern of Mtb-DHDPS with the best phyto-lead (SN00003544)

	H-bond formation			Salt bridge formation		
Residue	^1^Distance H-A	^2^Distance D-A	^3^Donor angle	Residue	^4^Distance	^5^Ligand group
THR54	3.62	3.96	102	ARG148	3.87	Carboxylate
THR55	1.91	2.8	154.89			
THR55	3.22	4.01	135.78			
LYS171	1.86	2.66	132.62			
SER252	2.45	3.31	148			
GLY256	2.19	3.04	138.74			

^1^distance between hydrogen and acceptor atoms, ^2^distance between donor and acceptor atoms, ^3^angle between donor, acceptor and hydrogen atoms, ^4^distance between centers of charge, ^5^functional group in the ligand providing the charge						

**Table 3:** Molecular interaction studies of best two PubChem hits akin to phytochemical lead and their comparison with known inhibitors

S. No.	Molecule ID	Scoring functions a^*^, b^$^, c^^^	H-bonding residues	Residues involved in molecular interactions
1	CID41032023	-10.998287	Thr54, Thr55, Arg148, Lys171, Gly256	Ala18, Met19, Val50, Gly53, Thr54, Thr55, Gly56, Leu111, Tyr143, Arg148, Lys171, Gly194, Asp195, Asp196, Val213, Met251, Gly256
		-140.286		
		-12.55		
2	CID54025334	-10.987286	Arg148, Lys171, Asp196, Ile211	Ala18, Thr54, Thr55, Tyr143, Arg148, Lys171, Gly194, Asp195, Asp196, Ile211, Ser212, Val213, Cys248, Met251, Ser252, Gly255, Gly256
		-140.244		
		-12.42		
3	CID557515	-6.09788	Thr55, Arg148, Lys171	Ala18, Thr54, Thr55, Tyr 143, Ile15, Arg148, Lys171, Gly194, Val213, Met251, Gly256,
		-117.856		
		-8.32		
4	CID12265924	-5.98698	Lys171, Asp195	Arg148, Lys171,Gly194, Asp195, Asp196, Ala197, Val213, Cys248, Met251, Ser252
		-116.927		
		-7.98		
5	CID10367	-7.527454	Arg148, Lys171, Asp195, Tyr143	Thr54, Thr55, Tyr 143, Ile145, Arg148, Lys171, Gly194, Asp195, Asp196, Ala197, Val 113, Ile 214, Met251
		-119.748		
		-9.34		
6	CID11390199	-7.217638	Thr55, Lys171, Asp195	Ala18, Thr54, Thr55, Tyr143, Arg148, Lys171, Gly194, Asp195, Asp196, Ile211, Ser212, Val213,
		-118.476		
		-9.22		
7	CID68297515	-6.112845	Arg148, Tyr143, Lys171	Thr54, Tyr143, Ile145, Gly147, Arg148, Lys171, Ala173, Gly194, Asp195, Asp196, Ile211, Ser212, Val213
		-117.909		
		-8.43		
8	PUB475318	-10.979285	Arg148, Lys171, Ser252	Ala18, Tyr 143, Ile145, Arg148, Lys171, Gly194,Asp195, Asp196, Ala197, Ile211, Ser212, Val213, Cys248, Met251, Ser252
		-140.236		
		-12.34		

^*^a: Docking Score of BioPredicta, ^$^b: MolDock Score of MVD, ^^^c: Free energy of binding of AutoDock				

**Table 4:** Binding pattern of Mtb-DHDPS with first potent PubChem compound (CID41032023)

Residues involved in H-bond formation				Salt bridge formation			Hydrophobic interaction			
Residue	^1^Distance H-A	^2^Distance D-A	^3^Donor angle	Residue	^4^Distance	^5^Ligand group	Residue	^6^Distance	^7^Ligand atom	^5 8^Protein atom
THR54	2.23	3.22	162.35	ARG148	3.7	Carboxylate	VAL213	3.62	2612	1830
THR55	2.14	2.83	128.61							
ARG148	2.08	2.63	111.23							
LYS171	2.91	3.73	137.7							
GLY256	2.26	3.2	153.38							

^1^distance between hydrogen and acceptor atoms, ^2^distance between donor and acceptor atoms, ^3^angle between donor, acceptor and hydrogen atoms, ^4^distance between centers of charge, ^5^functional group in the ligand providing the charge,^6^distance between interactions carbon atoms, ^7^ID of ligand carbon atom, ^8^ID of protein carbon atom										

**Table 5:** Binding pattern of Mtb-DHDPS with second potent PubChem compound (CID54025334)

Residues involved in H-bond formation				Salt bridge formation			Hydrophobic interaction			
Residue	^1^Distance H-A	^2^Distance D-A	^3^Donor angle	Residue	^4^Distance	^5^Ligand group	Residue	^6^Distance	^7^Ligand atom	^5,8^Protein atom
ASP195	3.14	3.79	127.71	LYS171	3.46	Carboxylate	THR54	3.91	2604	420
ASP196	1.7	2.63	149.15				VAL213	3.64	2606	1830
ILE211	2.46	3.2	132.63							
VAL213	3.15	3.62	109.58							
SER252	2.9	3.24	102.01							

^1^distance between hydrogen and acceptor atoms, ^2^distance between donor and acceptor atoms, ^3^angle between donor, acceptor and hydrogen atoms, ^4^distance between centers of charge, ^5^functional group in the ligand providing the charge, ^6^distance between interactions carbon atoms, ^7^ID of ligand carbon atom, ^8^ID of protein carbon atom										

**Table 6:** Binding pattern of known inhibitor of Mtb-DHDPS (PUB475318)

Residues involved in H-bond formation			
Residue	^1^Distance H-A	^2^Distance D-A	^3^Donor angle
ARG148	2.53	3.43	150.76
ARG148	2.43	3.12	128.42
LYS171	2.2	3.13	150.85
SER252	3.12	3.87	135.39

^1^distance between hydrogen and acceptor atoms, ^2^distance between donor and acceptor atoms, ^3^angle between donor, acceptor and hydrogen atoms			

## References

[1] McGuire S (2016). Adv Nutr.

[2] Lia DA (2015). J Thorac Dis.

[3] Viola RE (2010). J Amino Acid.

[4] Garg A (2010). BMC Bioinformatics.

[5] Jankute M (2015). Annu Rev Microbiol.

[6] Harer SL, Bhatia MS (2014). J Pharm Bioallied Sci.

[7] Khan MS (2011). Eur Rev Med Pharmacol Sci.

[8] Baig MH (2014). PLoS One.

[9] Baig MH (2015). Evid Based Complement Alternat Med.

[10] Akhtar S (2016). Interdiscip Sci Comput Life Sci.

[11] Sharma N (2016). Med Chem.

[12] Banerjee P (2015). Nucleic Acids Res.

[13] Lipinski CA (2004). Drug Discov Today Technol.

[14] Jayaram B (2012). BMC Bioinformatics.

[15] Sinha C (2015). Curr Top Med Chem.

[16] Salentin S (2015). Nucleic Acids Res.

[17] Snyder PW (2011). Proc Natl Acad Sci USA.

[18] Khan MK (2013). Pharmacogn Mag.

[19] Al-Khodairy (2013). Am J Bioinfo Res.

[20] Khan MKA (2015). J Chem Pharm Res.

